# Cranial and extracranial giant cell arteritis do not have different HLA-DRB1 and HLA-B association in Caucasian individuals

**DOI:** 10.1186/s13075-021-02659-9

**Published:** 2021-10-26

**Authors:** Diana Prieto-Peña, Sara Remuzgo-Martínez, Belén Atienza-Mateo, Raquel López-Mejias, Miguel Á. González-Gay, on behalf of the Spanish collaborative group for the study of large-vessel vasculitis, Fernanda Genre, Javier Gonzalo Ocejo-Vinyals, Alejandro Muñoz Jiménez, Francisco Ortiz-Sanjuán, Susana Romero-Yuste, Clara Moriano, Eva Galíndez-Agirregoikoa, Itziar Calvo, Norberto Ortego-Centeno, Noelia Álvarez-Rivas, José A. Miranda-Filloy, Irene Llorente, Ricardo Blanco, Oreste Gualillo, Javier Martín, Ana Márquez, Santos Castañeda, Iván Ferraz-Amaro

**Affiliations:** 1 Department of Rheumatology, Hospital Universitario Marqués de Valdecilla, Avenida Cardenal Herrera Oria s/n, 39011 Santander, Spain; 2 Research group on genetic epidemiology and atherosclerosis in systemic diseases and in metabolic bone diseases of the musculoskeletal system, IDIVAL, Santander, Spain; 3 School of Medicine, Universidad de Cantabria, Santander, Spain; 4 Cardiovascular Pathophysiology and Genomics Research Unit, School of Physiology, Faculty of Health Sciences, University of the Witwatersrand, Johannesburg, South Africa

To the editor,

We have read with interest the article published in *Arthritis Research & Therapy* by Kushimoto et al. suggesting that the HLA-B*52 allele may indicate the presence of diffuse extracranial large vessel vasculitis (LVV) in patients with giant cell arteritis (GCA) [1]. GCA is the prototype of LVV that affects people over 50 years of age, especially those of European descent [2]. Unlike Takayasu arteritis, a LVV that predominantly affects people under 50 years of age, GCA is rare in Asian countries [3]. For years, GCA was considered a cranial disease. However, the use of imaging techniques has shown that it also affects large extracranial vessels [4]. In this sense, in some cases, GCA has a pure extracranial phenotype. These patients are usually younger and have polymyalgia rheumatica (PMR) more frequently than those with a classic pattern of cranial GCA [5].

There is a genetic component in the pathogenesis of GCA [6]. Different studies point to the influence of genes located in the MHC region, in particular the HLA-DRB1*04 alleles [7]. Since the pattern of vascular involvement in Takayasu arteritis often resembles that found in patients with extracranial GCA, and the genetic contribution to the pathogenesis of the disease is mainly mediated by the HLA-B*52 allele [8], we wondered if in Caucasian individuals the association of HLA with GCA may differ according to the predominant pattern of the disease. For this reason, we evaluated a series of 105 Spanish patients older than 50 years with extracranial LVV without cranial manifestations and compared them with a series of 184 patients with biopsy-proven GCA with typical cranial manifestations of the disease, who did not present features of extracranial involvement. We confirmed in our population the previously reported association of GCA with HLA-DRB1*04, in particular with the HLA-DRB1*04:01 allele, regardless of the clinical phenotype [9]. In a further step, we looked for possible differences in the *HLA-B* locus. However, we did not find *HLA-B* differences between these two subgroups of Spanish patients with GCA that showed a similar association with the HLA-B*15 allele, mainly due to HLA-B*15:01. Furthermore, we found that the presence of HLA-B*15:01 and HLA-DRB1*04:01 increases the risk of developing both cranial and extracranial LVV-GCA in our population [10].

Kushimoto et al. evaluated 40 Japanese patients over 50 years of age who were categorized as elderly-onset LVV (EOLVV) that showed diffuse vascular lesions similar to those found in extracranial LVV-GCA, 13 of them also had temporal artery involvement, while 27 did not [1]. The 27 patients with isolated extracranial EOLVV were classified into two groups: 11 and 16 patients with and without PMR. The 11 extracranial EOLVV patients with PMR and the 13 patients with both cranial and extracranial involvement formed a cluster of patients who were HLA-B*52 positive [1]. However, we could not demonstrate a higher prevalence of the HLA-B*52 allele in Spanish patients with extracranial GCA [10]. In this regard, only 2 of 105 Spanish patients with extracranial-LVV carried the HLA-B*52 allele. This frequency was similar to that observed in patients with cranial GCA (4 of 184) and healthy controls (15 of 486). Accordingly, HLA-B*52 cannot be considered as a marker for extracranial LVV in Caucasian individuals over 50 years of age.

It is possible that different genetic backgrounds and the influence of other genes located outside the MHC region may explain the differences in the incidence and clinical expression of LVV in Caucasian individuals compared to those in Asian countries.

## Data Availability

All data generated or analyzed during this study are included in this published article.

## Abbreviations

GCA: Giant cell arteritis
LVV: Large vessel vasculitis
PMR: Polymyalgia rheumatica

## References

[1] Kushimoto K, Ayano M, Nishimura K, Nakano M, Kimoto Y, Mitoma H (2021). H. HLA-B52 allele in giant cell arteritis may indicate diffuse large-vessel vasculitis formation: a retrospective study. Arthritis Res Ther.

[2] Gonzalez-Gay MA, Vazquez-Rodriguez TR, Lopez-Diaz MJ, Miranda-Filloy JA, Gonzalez-Juanatey C, Martin J (2009). Epidemiology of giant cell arteritis and polymyalgia rheumatica. Arthritis Rheum.

[3] González-Gay MA, García-Porrúa C (2001). Epidemiology of the vasculitides. Rheum Dis Clin North Am..

[4] González-Gay MA, Prieto-Peña D, Calderón-Goercke M, Atienza-Mateo B, Castañeda S (2020). Giant cell arteritis: more than a cranial disease. Clin Exp Rheumatol..

[5] González-Gay MA, Prieto-Peña D, Martínez-Rodríguez I, Calderon-Goercke M, Banzo I, Blanco R, Castañeda S (2019). Early large vessel systemic vasculitis in adults. Best Pract Res Clin Rheumatol..

[6] Carmona FD, González-Gay MA, Martín J (2014). Genetic component of giant cell arteritis. Rheumatology (Oxford)..

[7] Carmona FD, Mackie SL, Martín JE, Taylor JC, Vaglio A, Eyre S, Bossini-Castillo L (2015). Am J Hum Genet..

[8] Saruhan-Direskeneli G, Hughes T, Aksu K, Keser G, Coit P, Aydin SZ (2013). Identification of multiple genetic susceptibility loci in Takayasu arteritis. Am J Hum Genet..

[9] Prieto-Peña D, Remuzgo-Martínez S, Ocejo-Vinyals JG, Atienza-Mateo B, Muñoz-Jiménez A, Ortiz-Sanjuán F (2020). Cranial and extracranial giant cell arteritis share similar HLA-DRB1 association. Semin Arthritis Rheum..

[10] Prieto-Peña D, Remuzgo-Martínez S, Ocejo-Vinyals JG, Atienza-Mateo B, Genre F, Muñoz-Jimenez A (2021). Clin Exp Rheumatol..

